# *Aspergillus fumigatus* cerebral abscess following hemodialysis: A case report

**DOI:** 10.32598/CMM.2023.1323

**Published:** 2022-12

**Authors:** Tanu Sagar, Lata Sheoran, Ajay Prajapati, Bembem khuraijam, Partha Pratin Jana, P. N Pandey, Sonal Saxena

**Affiliations:** 1 Department of Microbiology, Maulana Azad Medical College, New Delhi, India; 2 Department of Neurosurgery, Lok Nayak Hospital, New Delhi, India; 3 Department of Pathology, Maulana Azad Medical College, New Delhi, India

**Keywords:** *Aspergillus fumigatus*, Cerebral abscess, Hemodialysis

## Abstract

**Background and Purpose:**

Cerebral aspergillosis is a notorious disease that causes rapid clinical deterioration and carries a poor prognosis. Therefore, it requires timely diagnosis and prompt management.

**Case Report:**

This study reports a case of fungal cerebral abscess in a 26years old man following hemodialysis,2 months afterdengue-induced acute kidney disease. *Aspergillus fumigatus* was recovered from a brain abscess specimen that was subjected to a parietal craniotomy. The patient was successfully treated with oral Voriconazole 400mg BD for 2 days, followed by 200 mg BD for 3months.

**Conclusion:**

Hemodialysis patients are at high risk offungal infections due to the frequent use of catheters or the insertion of needles to access the bloodstream. Therefore, a high index of suspicion of fungal infection is required in patients with hemodialysis by the clinician for early diagnosis and treatment.

## Introduction

Fungal diseases are on the rise due to an ongoing increase in transplant surgeries, immune-suppression states, and the use of antibiotics. It has a more aggressive and catastrophic course, compared to the bacterial and protozoan cerebral abscess, with a high mortality rateranging from 80% to 99% [ 1 ]. The brain can be infected with fungus through hematogenous dissemination from a distant source, such as the lung, local extension from sinonasal, orbital, and spinal infections, or direct implantation after trauma [ 2 ]. This study presents a case of successfully treated *Aspergillus fumigatus* cerebral abscess in a 26 years old man following hemodialysis.

## Case report

A 26-year-old male patient was admitted to our hospital with chief complaints of a diffuse, throbbing headache with severe intensity for the past 1 month. A week before admission, he had suffered from frequent episodes of non-projectile, non-blood-stained, and non-bilious vomiting. No history of sinusitis, mastoiditis, lung infection, head injury, steroid intake, or COVID-19 infection was reported. However, 2months before the admission, he was admitted and treated for dengue fever in a local hospital. 

During his admission, he developed sepsis-induced acute kidney injury and was on maintenance hemodialysis (twice a week) for 2 months thereafter. After 2 weeks of hemodialysis, the patient developed headaches and vomiting with altered sensorium. On examination, he was conscious and had pallor and pedal edema with no other systemic abnormalities. Glasgow coma scale score was E4V5M6 and no sensorimotor deficit was noted. 

On investigations, hemoglobin was 7.2 g/dL, total leucocyte count was 10,600 cells/cu.mm with (neutrophils: 79% and Lymphocytes: 15%), serum creatinine was 1.65 mg/dl and serum urea was 73mg/dl. The patient was then investigated, and the Computed Tomography scan (CT)of the head revealed a hypodense lesion in the left parietal region with ill-defined perilesional hypodensity showing meningoencephalitis.

**Figure 1:**
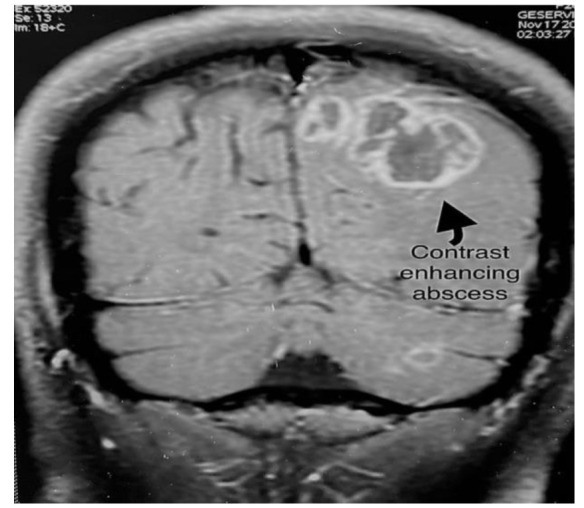
Magnetic resonance imagingscan showing multiple ring enhancement lesions

Magnetic resonance imaging (MRI) brain showed multiple ring enhancement lesions of altered signal intensity and variable sizes in the left cerebral hemispheres(Figure 1). The features were consistent with the infective or inflammatory micro-abscesses.

**Figure 2:**
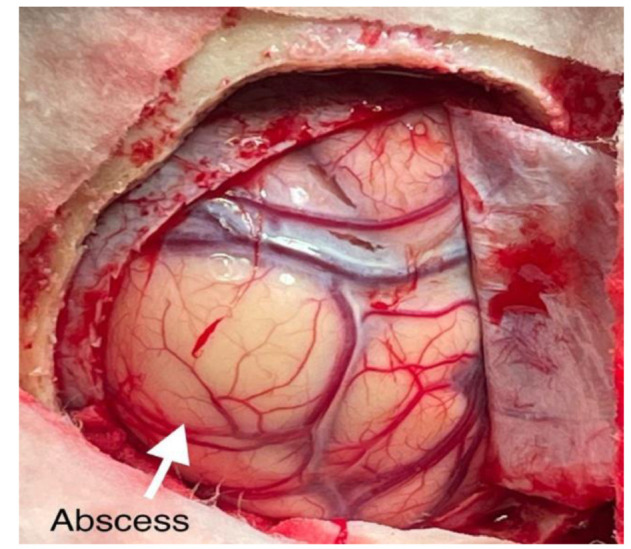
Intra-operative view of the cerebral abscess

A provisional diagnosis of parietal brain abscess with meningoencephalitis (fungal or tubercular) was made and the patient was started on parenteral ceftriaxone. A left parietal craniotomy was performed with complete excision of the abscess (Figure 2), and the specimen was sent for microbiological and histopathological investigations. 

**Figure 3:**
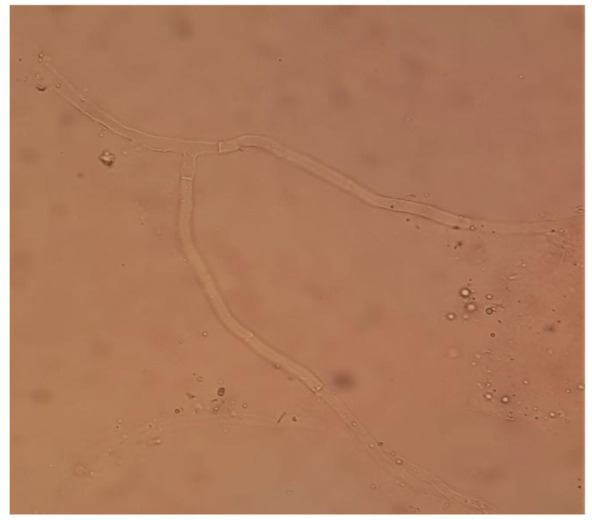
Potassium hydroxide mountshowing hyaline fungal septate hyphae

**Figure 4:**
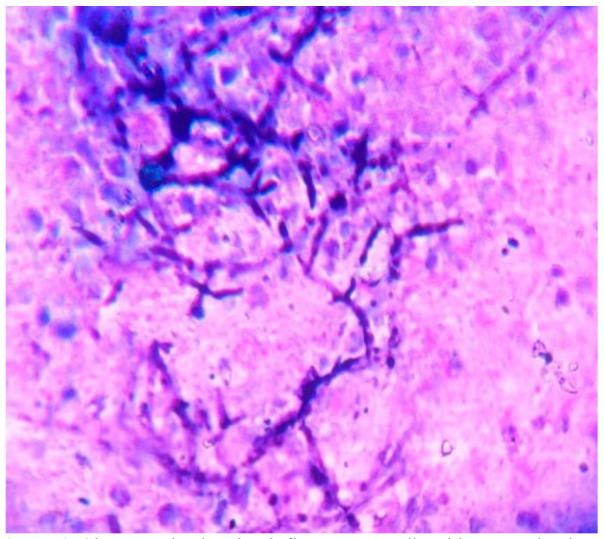
Giemsa stain showing inflammatory cells with septate hyphae

**Figure 5:**
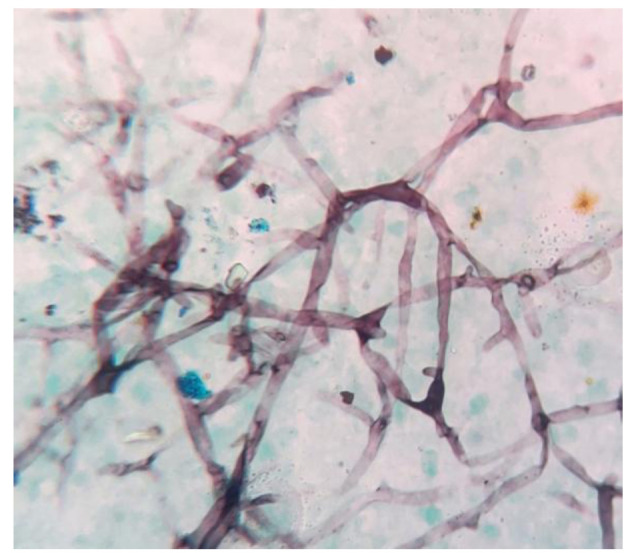
Grocott’s methenamine silver stain illustrates septate hyphae branching at acute angles

Potassium hydroxide mount(KOH)showed hyaline septate branching fungal hypha (Figure3). Histopatho-logical examination using special stains (Giemsa and Grocott’s methenamine silver) also showed the presence of numerous septate fungal hyphae with inflammatory cells(figures 4 and 5).The staining did not reveal any acid-fast structures in acid-fast bacilli. Moreover, the routine culture for bacteria did not show any growth. In addition, viral markers (human immunodeficiency virus, Hepatitis B antigen, anti-hepatitis C antibodies ) were negative. 

**Figure 6:**
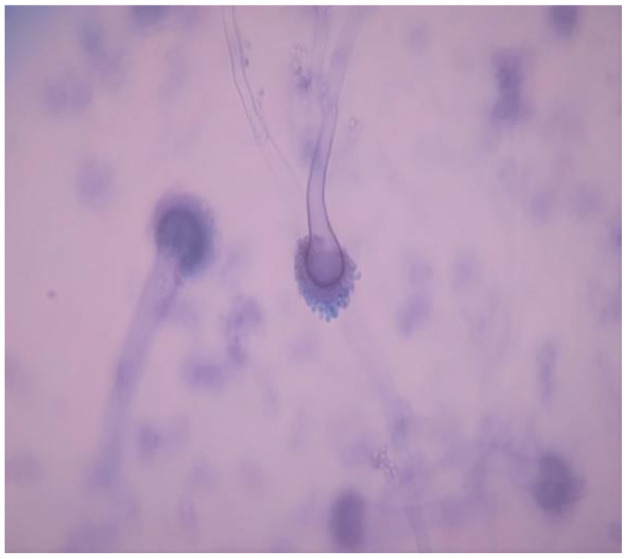
Lacto-phenol cotton bluemount showing *Aspergillus fumigatus*

Based on the above-mentioned findings, a definite diagnosis of fungal brain abscess was made and the *Aspergillus* species was the first differential diagnosis. The patient was started with oral voriconazole 400mg BD for 2 days, followed by 200 mg BD for 3months. On the third day after surgery, the patient remained stable and was discharged. After 10 days, the fungal culture showed the growth of greenish-colored colonies on brain heart infusion biphasic medium(manufactured by HiMedia, India), which were identified as *A.fumigatus* on Lactophenol cotton blue mount (Figure 6) that was confirmed on MALDI-TOF (Bruker).

It should also be mentioned that the culture for *Mycobacterium tuberculosis* was negative after 8 weeks of incubation. The patient had fully recovered from his neurological symptoms and was able to start his normal daily activities 3months after being discharged. 

## Discussion

The brain is resistant to fungal infections due to the relatively impermeable blood-brain barrier. *Cladophialophorabantiana* (neurotropic fungus) is the predominant causative agent of fungal cerebral abscess followed by Aspergillus [ 1 ]. Neuroaspergillosis accounts for 5% of all cranial infections[ 3 ]. These infections are difficult to treat and often lead to mortality[ 1 , 4 ]. The diagnosis is mostly made in the terminal stage or after the death of the patient. 

The most common pathological signs in neuro-aspergillosis include hemorrhage, infarctions, and abscesses. It is commonly seen in patients with immunocompromised conditions.However, cases in immunocompetent individuals have also been reported to occur after direct trauma, through the use of contaminated medical devices and intravenous drug misuse[ 5 - 7 ].

Neuroimaging techniques, such as contrast CT scan or MRI are important diagnostic toolsforthe identification of brain abscesses that help to differentiate a fungal cerebral abscess from a bacterial abscess. A fungal cerebral abscess presents with a typical finding of a hypodense lesion with a contrast-enhancing ring in a CT scan or MRI scan [ 8 ]. Unlike bacterial abscesses, which are usually solitary lesions, fungal abscesses are more likely to be numerous. Focal lesion formation is the most common finding seen in patients with CNS aspergillosis [ 9 ]. Therefore, neuroimaging plays a significant role in the diagnosis and therapeutic decision-making for CNS infections.

Despite the availability of neuroimaging techniques, the establishment of a definitive diagnosis of brain lesions is often difficult; and invasive strategies, such as stereotactic brain biopsy, may be required. Direct microscopy, culture, and histopathology from a sterile specimen are definitive for the diagnosis of invasive fungal infections. Non-culture-based approaches, like galactomannan(GM) and polymerase chain reaction(PCR), have only been verified for use in serum and bronchoalveolar lavage for the diagnosis of invasive aspergillosis[ 10 ]. Anti-fungal medications are generally harmful and should not be given on a long-term basis to any patient without a definitive diagnosis of fungal infection; hence,diagnosticimaging should not be used in place of pathological or microbiological diagnosis.

*A.fumigatus* is most frequently reported in immunosuppressed patients while A.flavusis often reported in immunocompetent patients. Other species, such as *A.niger*, *A.terreus*, *A.versicolor*, *A.nidulans*, *A.glaucus*, *A.clavatus*, *A.oryzae*, *A.sydowii*, and *A.ustus* are less often determined[ 11 , 12 ]. Apart from being angioinvasive in nature, *Aspergillus* spp. also produce mycotoxins that inhibit phagocytosis and reduce conidia opsonization during the invasion [ 13 ]. Along with this, Mycotoxins have the potential to change the integrity of the blood-brain barrier and harm and kill the brain cells (i.e., neurons, astrocytes, and microglia)[ 13 ].

It should be mentioned that the treatment of cerebral aspergillosis has been challenging. The CNS is protected by various barriers (blood-brain barrier), which restricts the entrance of plausible harmful substances. For decades, Amphotericin B (AmB) was the only therapeutic drug used for the treatment of cerebral abscesses; however, it was found to be less effective. Amphotericin B is a large molecule, less efficient to cross the blood-brain barrier, and therefore, less likely to reach adequate concentrations in the CNS (with amphotericin B deoxycholate, brain tissue concentrations ranged from 0.2 to 5.8 mg/g, and with liposomal amphotericin B, concentrations ranged from undetectable to 1.6 mg/g) [ 14 ].

Moreover, the use of amphotericin B is limited due to known fungal resistance and severe nephrotoxicity. Similarly, echinocandins (i.e., micafungin, caspofungin, and anidulafungin) also have poor CNS penetration due to their large size (1140 to 1292 Da). In one study, when a daily dose of 300 mg micafungin was administered to patients with cerebral aspergillosis, they exhibited a modest micafungin level (50.017 mg/ml) in the CSF fluid [ 15 ]. The lipophilic nature of itraconazole and posaconazole drugs allows better penetration into the CNS; however, investigations have revealed insignificant amounts of these compounds in the CNS[ 16 ].

Voriconazole is a second-generation triazole drug with good antifungal activity against many fungal pathogens. It has a small molecule and is known to have a strong blood-brain barrier penetration, making it a prospective treatment option for cerebral aspergillosis. It is also available in oral formulation, besides intravenous formulation with an oral bioavailability of greater than 90% [ 17 ]. Voriconazole is a broad-spectrum antifungal drug that is usually well-tolerated and has possible side effects that resolve spontaneously after the end of the treatment. As a result, current recommendations suggest using voriconazole (oral or intravenous) as the first-line treatment for CNS aspergillosis[ 18 ].

Aside from antifungal medicines, neurosurgical treatments can be beneficial depending on the circumstances of the patient, such as the location and extent of the brain lesions. Stereotactic biopsy/ aspiration is a minimally invasive procedure that is performed when the abscess is multiple, deep-seated, and located in an eloquent region of the brain or the general condition of the patient precludes major surgeries [ 19 ].Craniotomies/radical surgeries are performed for the abscesses which are in relatively accessible regions of the brain. According to the findings of a previous study, surgical excision of focal CNS aspergillosis lesions reduces the death rate from 64% to 39%[ 20 ]. Therefore, anti-fungal treatment along with surgical intervention plays an important role in the recovery of patients.

In our case, the likely cause of infection was a medical device intervention, which our patient had throughout his hemodialysis sessions, as no other site of aspergillosis was detected. He had no history of immunocompromised conditions, such as diabetes, prolonged corticosteroid use, or neutropenia. Although, he was on hemodialysis since 2 months ago due to dengue-induced acute kidney disease.

Hemodialysis patients require frequent use of catheters or insertion of needles to access the bloodstream, and therefore, are at high risk of infection. Moreover, these patients have weakened immune systems due to uremia, which further increases their risk of infection [ 21 ]. Fortunately, this patient was young and had no other history of prolonged illness. He showed clinical improvement with surgical intervention followed by the oral administration of voriconazole.

## Conclusion

Fungal cerebral abscess is rare but leads to high morbidity and mortality if diagnosis and treatment are delayed. The clinician should keep a high index of suspicion for these lesions and the specimen from the CNS should be subjected to fungal cultures in addition to cultures for bacteria. Isolation of pathogens on culture further helps to determine the antifungal resistance. Correct identification of the agent is necessary to effectively treat brain abscesses as early as possible. Therefore, a multidisciplinary approach is required for the management of the patient with brain infections and should involve neuroradiology, infectious diseases, neurosurgery, and critical care medicine.

## Acknowledgments

The authors would like to express their gratitude to Mr. Koshel for his technical help.

## Authors’ contribution

T.S., L.S., A.P., and S.S. conducted the study. T.S., L.S., B.K., and P.P.J. made the mycological and histopathological identification of the isolated strain. L.S., A.P., and P.P.J. collected the clinical data. T.S., L.S., P.N.P., and S.S. created the final draft of the manuscript. All authors read and approved the final manuscript.

## Conflicts of interest

The authors declare no conflicts of interest.

## Financial disclosure

The research received no specific grant from any funding agency.

## Ethical considerations

Written informed consent was obtained from the patient.
